# The Joint Influence of Gender and Amount of Smoking on Weight Gain One Year after Smoking Cessation

**DOI:** 10.3390/ijerph110808443

**Published:** 2014-08-18

**Authors:** Isabella Locatelli, Tinh-Hai Collet, Carole Clair, Nicolas Rodondi, Jacques Cornuz

**Affiliations:** 1 Department of Ambulatory Care and Community Medicine, University of Lausanne, Rue du Bugnon 44, CH-1011 Lausanne, Switzerland; E-Mails: tinh-hai.collet@chuv.ch (T.-H.C.); carole.clair@hospvd.ch (C.C.); jacques.cornuz@chuv.ch (J.C.); 2 Institute of Social and Preventive Medicine, University of Lausanne, Route de la Corniche 10, CH-1010 Lausanne, Switzerland; 3 Service of Endocrinology, Diabetes and Metabolism, University Hospital of Lausanne, Rue du Bugnon 46, CH-1011 Lausanne, Switzerland; 4 Department of General Internal Medicine, Inselspital, Bern University Hospital, CH-3010 Bern, Switzerland; E-Mail: nicolas.rodondi@insel.ch

**Keywords:** weight gain, smoking cessation, overall duration of abstinence, weight gain risk factors, sex interaction

## Abstract

Weight gain is often associated with smoking cessation and may discourage smokers from quitting. This study estimated the weight gained one year after smoking cessation and examined the risk factors associated with weight gain in order to identify socio-demographic groups at higher risk of increased weight after quitting. We analyzed data from 750 adults in two randomized controlled studies that included smokers motivated to quit and found a gradient in weight gain according to the actual duration of abstinence during follow-up. Subjects who were abstinent for at least 40 weeks gained 4.6 kg (SD = 3.8) on average, compared to 1.2 kg (SD = 2.6) for those who were abstinent less than 20 weeks during the 1-year follow-up. Considering the duration of abstinence as an exposure variable, we found an age effect and a significant interaction between sex and the amount of smoking before quitting: younger subjects gained more weight than older subjects; among light smokers, men gained more weight on average than women one year after quitting, while the opposite was observed among heavy smokers. Young women smoking heavily at baseline had the highest risk of weight gain after quitting.

## 1. Introduction

Cigarette smoking is the leading preventable cause of mortality and is responsible for nearly six million deaths worldwide [1]. Thus, smoking cessation is widely recommended. However, weight gain after smoking cessation is a major drawback [2,3,4,5] and often discourages smokers from quitting, or may even precipitate a relapse [6,7]. Although the existence of weight gain after smoking cessation is widely recognized, the estimation of its magnitude is variable. Reviews performed at the end of the 1980s estimated a weight gain of 1.8 to 3.6 kg one year after smoking cessation [8,9]. A recent meta-analysis based on 62 intervention studies completed in the 1990s and 2000s estimated that 4.7 kg are gained one year after smoking cessation [10].

One reason for this heterogeneity is certainly the use of different definitions of abstinence. Subjects involved in a program for smoking cessation may quit and relapse several times during the follow-up [11,12,13]. In early studies, “point prevalence” abstinence was commonly adopted, which is defined as not smoking in the week prior to a certain time point without considering previous relapses, whereas more recent and stringent studies have used “continuous” abstinence, which is defined as not smoking from baseline to follow-up [14]. The use of point prevalence abstinence probably leads to underestimating the weight gain due to smoking cessation [4,15]. However, adoption of the stringent continuous abstinence definition may lead to considering subjects relapsing for very short periods as not being abstinent, while they are susceptible to weight gain almost as much as continuously abstinent subjects.

Weight gain after smoking cessation may also vary according to smokers’ individual characteristics. Young and heavy smokers have been found to be at higher risk of weight gain in several studies [16,17,18]. The gender effect is less clear in the literature; some studies have reported that women gain more weight than men [17,19], whereas others have stated the opposite [20,21] or found non-significant differences [22].

In the present study, we hypothesized the existence of a dose-response relationship between duration of smoking abstinence and weight gain [23]. We studied weight gain one year after smoking cessation according to the overall time spent abstinent during follow-up, which was treated as an exposure variable. In addition, accounting for the exposure to abstinence, we also aimed to identify individual risk factors associated with weight gain following smoking cessation.

## 2. Methods

### 2.1. Data

We used individual participant data from two recent randomized controlled trials (RCTs) assessing novel interventions to help smokers quit. The first RCT (PHASMO; *n* = 481) evaluated the impact of a program of moderate intensity physical activity on cessation rates after one year [11,12]. The second RCT (CAROSS; *n* = 536) assessed the motivational effect of carotid plaque screening by ultrasound [13]. The interventions did not show any significant effects on smoking cessation rates, allowing us to consider data from each RCT as a cohort, regardless of the RCT-specific randomization group. Merging the two cohorts was feasible considering their multiple similarities: (a) both involved long-term smokers motivated to quit, (b) both had a 1-year follow-up, (c) both had a control program of intensive counseling and nicotine replacement therapies, and (d) the subjects in both studies were followed at the same site (University of Lausanne) by the same research team specifically trained in smoking cessation counseling, allowing a homogenous counseling approach. This combined data analysis can be regarded as an individual participant data meta-analysis [24], though we were not interested in the main outcome of the source RCTs, *i.e.*, smoking cessation rate, but in a secondary pooled data analysis of the weight gain associated with smoking cessation.

### 2.2. Outcomes and Inclusion/Exclusion Criteria

Weight was measured at baseline and the 1-year follow-up visit in the CAROSS study. In PHASMO, weight measures were available at each follow-up visit (every week during the first 10 weeks and at the 6- and 12-month follow-up). In order to maximize the information coming from the two RCTs, the main outcome variable was defined as the absolute weight difference between baseline and the last follow-up visit. Therefore, we included only subjects with weight measurements available at the two visits (PHASMO, *n* = 473; CAROSS, *n* = 422).

For consistency with previous analyses of weight gain on PHASMO data [12] and to assess the effect of smoking cessation on weight, we only included subjects who made an attempt to quit during follow-up (PHASMO, *n* = 419; CAROSS, *n* = 333). The mean weight gain of subjects who never attempted to quit (0.3 kg) was not significantly different from the 1-year mean weight gain of the general population, as estimated by two studies [19,25].

**Figure 1:**
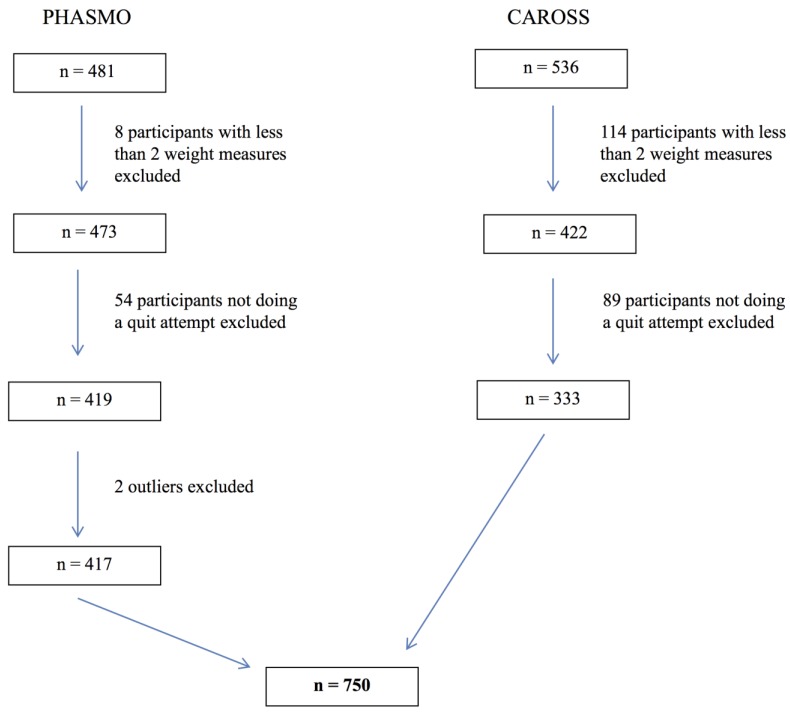
Flow chart of participants from both randomized controlled trials.

Notes: Abbreviations: CAROSS, The Impact of CAROtid plaque Screening on Smoking cessation [13]; PHASMO, PHysical Activity as an aid for SMOking cessation [11,12].

We excluded one outlier regarding weight gain in PHASMO (an individual who lost 43 kg during the 1-year follow-up) and one outlier of the same dataset who attended his last visit after 2 years (much longer than the standard 1-year follow-up) (Figure 1). As a secondary outcome, we also considered the difference in weight between baseline and the last attended visit as a percentage of the baseline weight (relative weight difference).

### 2.3. Covariates and Assumptions

We considered the following baseline variables as potential predictors of weight change after smoking cessation: age, sex, education level (defined as the number of completed years of school), and baseline smoking intensity, measured with the number of cigarettes smoked per day before quitting [12,13].

Both RCTs [12,13] consistently defined abstinence at each visit as self-reported smoking cessation validated by a carbon monoxide (CO) concentration in exhaled air <10 ppm; subjects with a CO concentration >10 ppm or who reported smoking were considered to have relapsed. Individual abstinence trajectories were variable in both RCTs, with many subjects quitting and relapsing more than once during follow-up. Following an approach similar to the one adopted by Aubin *et al.* [23], we considered the individual overall duration of abstinence between baseline and the last follow-up visit as an exposure variable in determining weight gain. The overall duration of abstinence was calculated by summing the durations of all abstinence periods during follow-up. We defined the first quit date as the mid-date between the last visit with smoking status and the first visit with abstinence status; relapse dates and higher order quit dates were defined as the midpoint of two consecutive visits involving a change in status (abstinent to relapse, or relapse to abstinent).

Both RCTs were scheduled for 1-year follow-up. However, an important percentage of drop-out was observed, especially in the PHASMO RCT. Assuming that subjects lost to follow-up relapsed, as recommended for smoking cessation analyses [14], exclusion of these subjects would lead to overestimation of the weight gain for PHASMO. We included these subjects and assumed that their weight remained stable after they left the study and relapsed. This assumption was in line with the non-significant weight change observed by Prod’hom *et al.* [12] with the PHASMO data for relapse periods occurring during the first 9 weeks of follow-up. Similar results were obtained by applying Prod’hom *et al.* [12] model to the entire 1-year follow-up. The assumption was also validated empirically by a strong similarity between the weight gain distributions in the PHASMO and CAROSS studies (Section 3). Therefore, we adopted a “Last Observation Carried Forward” (LOCF) approach for missing values in the overall duration of abstinence and weight gain [26,27]. With these assumptions, the overall duration of abstinence at the last attended visit could be considered a proxy for the 1-year cumulative duration of abstinence, and the absolute weight difference at the last attended visit could be considered as a proxy for the 1-year absolute weight difference.

### 2.4. Statistical Analysis

We compared individual characteristics between the two RCTs using the t-test for continuous and symmetric variables, Mann-Whitney test for skewed variables, and z-test for dichotomous variables. Bimodal variables (duration of follow-up and cumulative duration of abstinence) were categorized and compared using the χ^2^ test.

All potential predictors of weight gain were introduced in a multivariable linear regression model. Variables exhibiting a right asymmetry were transformed as needed to achieve better symmetry and normality. A dichotomous variable was introduced in order to control for the particular RCT (PHASMO or CAROSS) a subject was from, as recommended by Riley *et al.* [24] for an individual participant data meta-analysis. We also introduced into the model an indicator of the RCT-specific randomization group in order to control for any potential differences in weight gain between intervention and control groups. All pair-wise interactions between covariates were tested, but only significant interactions were kept in the final model.

## 3. Results

The two RCT datasets included 750 subjects: 417 from PHASMO and 333 from CAROSS (Figure 1). The proportion of women (43.6%) and education level were not significantly different between trials (Table 1). PHASMO subjects were significantly younger than CAROSS subjects (mean 42.7 years *vs.* 51.4 years, *p* < 0.001) and smoked more cigarettes at baseline (median 25 *vs.* 20 per day, *p* < 0.001). The distribution of individual follow-up duration differed between RCTs (*p* < 0.001): in CAROSS almost all subjects (99%) were followed for more than 40 weeks, whereas in PHASMO 32% of subjects were followed fewer than 40 weeks. Despite the difference in individual follow-up duration, the RCTs had similar distributions of the cumulative duration of abstinence (*p* = 0.50) with 52% of subjects being abstinent fewer than 20 weeks (Table 1).

**Figure 2:**
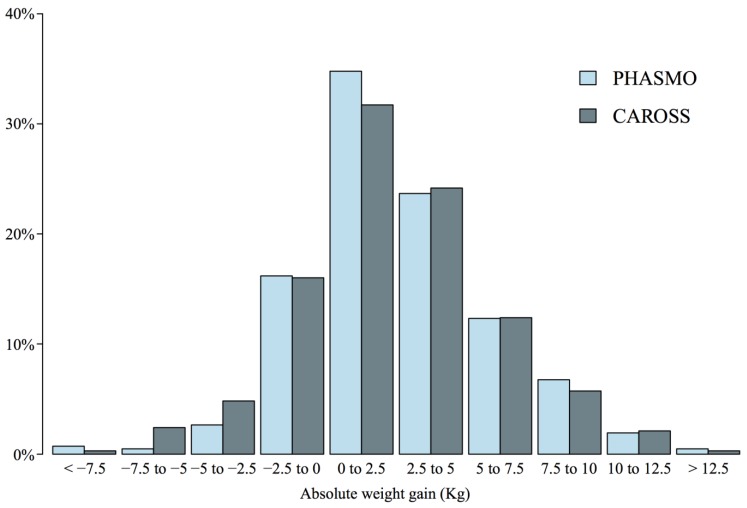
Distribution of absolute weight gain (kg) at one year, by randomized clinical trial. Abbreviations: CAROSS, The Impact of CAROtid plaque Screening on Smoking cessation; PHASMO, PHysical Activity as an aid for SMOking cessation.

The mean absolute weight difference at the last follow-up visit (our proxy for 1-year weight gain) was 2.6 kg (SD = 3.7) with no significant difference between RCTs (*p* = 0.31, Table 1). Weight gain distributions were similar in both RCTs (*p* = 0.32), and 57% of subjects gained between 0 and 5 kg (33% between 0 and 2.5 kg, Figure 2). Weight gain increased according to the cumulative duration of abstinence, from 1.2 kg (SD = 2.6) for abstinence <20 weeks to 4.6 kg (SD = 3.8) for abstinence >40 weeks (*p* for trend <0.001, Table 2). The same trend was observed for relative weight gain.

**Table 1:** Individual characteristics.

Characteristics	PHASMO (*n* = 417)	CAROSS (*n* = 333)	*p*-value for Difference	Overall (*n* = 750)
**Age**, mean (SD)	42.7 (9.8)	51.4 (9.8)	<0.001 *	46.6 (9.8)
**Women**, No. (%)	181 (43.4)	146 (43.8)	0.90 ^†^	327 (43.6)
**Education level**, No. (%) ^‡^			0.09 ^§^	
9 years	49 (12.3)	33 (10.6)		82 (11.5)
12 years	223 (55.9)	209 (67.2)		432 (60.9)
14 years	72 (18.0)	32 (10.3)		104 (14.6)
16 years	55 (13.8)	37 (11.9)		92 (13.0)
**No. cigarettes per day at baseline**, median (IQ range)	25 (20–30)	20 (17–30)	<0.001 ^§^	22 (20–30)
**Follow-up**, No. (%) ^||^			<0.001	
<20 weeks	110 (26.4)	0 (0.0)		110 (14.7)
20–40 weeks	25 (6.0)	2 (0.6)		27 (3.6)
>40 weeks	282 (67.6)	331 (99.4)		613 (81.7)
**Cumulative duration of abstinence**, No. (%)			0.50 ^¶^	
<20 weeks	212 (50.8)	174 (52.3)		386 (51.5)
20–40 weeks	50 (12.0)	31 (9.3)		81 (10.8)
>40 weeks	155 (37.2)	128 (38.4)		284 (37.7)
**Absolute weight gain**, kg ^#^, mean (SD)	2.7 (3.6)	2.4 (3.8)	0.31 *	2.6 (3.7)
**Relative weight gain**, %, mean (SD)	3.8 (5.1)	3.2 (5.0)	0.11 *	3.6 (5.0)

Notes: Abbreviations: SD = standard deviation; IQ = interquartile. * *t*-test. **^†^** test for the difference between two proportions. **^‡^** *n* = 710 (40 missing values). **^§^** Mann-Whitney test. **^||^** *n* = 749 (1 missing value). **^¶^** Chi^2^ test. **^#^** Weight in kilograms (kg), multiply by 2.2 to convert to lbs.

**Table 2:** Absolute and relative weight gain for categories of overall duration of abstinence.

Cumulative Duration of Abstinence, No. (%)	Absolute Weight Gain, kg, Mean (SD)	Relative Weight Gain, %, Mean (SD)
<20 weeks	1.2 (2.6)	1.7 (3.7)
20–40 weeks	2.0 (4.4)	3.1 (5.9)
>40 weeks	4.6 (3.8)	6.2 (5.2)

The model was then adjusted to include all available risk factors potentially related to weight gain. In the adjusted model (*R*^2^ = 0.21), neither the original RCT (PHASMO or CAROSS) nor the intervention group were significantly related to weight gain (Table 3). The education level was not significantly associated with weight gain and was dropped from the model because of the number of missing values for this variable (*n* = 40 missing values). Significant predictors of weight gain were the cumulative duration of abstinence, age, sex, and the interaction between sex and the number of cigarettes smoked at baseline. The model estimated a mean weight gain of 1.1 kg (95% CI 0.3 to 1.9) for men aged 47 years (the cohort mean) who smoked 10 cigarettes at baseline (the cohort minimum) and had <20 weeks of smoking abstinence during follow-up. Similar to the unadjusted analysis (Table 2), weight gain increased significantly with longer duration of abstinence (*p* for trend <0.001, Table 3). 

**Table 3:** Adjusted linear regression model of absolute and relative weight gain (*R*^2^ = 0.21).

Characteristics	Outcome = Absolute Weight Gain (kg)		Outcome = Relative Weight Gain (%)	
	Estimate (95% CI)	*p*-value	Estimate (95% CI)	*p*-value
**Intercept**	1.1 * (0.3; 1.9)	0.005	1.4 (0.3; 2.5)	0.02
**Study**				
CAROSS	Reference		Reference	
PHASMO	−0.1 (−0.7; 0.4)	0.66	0.1 (−0.7; 0.8)	0.89
**Group**				
Control	Reference		Reference	
Intervention	−0.1 (−0.5; 0.4)	0.81	−0.2 (−0.8; 0.5)	0.65
**Abstinence**				
<20 weeks	Reference		Reference	
20–40 weeks	0.9 ^†^ (0.1; 1.7)	0.02	1.5 (0.4; 2.6)	0.009
>40 weeks	3.4 ^‡^ (2.9; 3.9)	<0.001	4.6 (3.9; 5.3)	<0.001
**Age (10 years)**	−0.3 (−0.6; 0.0)	0.02	−0.4 (−0.8; −0.1)	0.02
**Sex**				
Men	Reference			
Women	−1.2 ^§^ (−2.2; −0.2)	0.02	−1.0 (−2.4; 0.4)	0.16
**√NCB ^||^**	0.2 ^#^ (−0.2; 0.5)	0.32	0.2 (−0.3; 0.6)	0.52
**Sex √NCB**	0.5 ** (0.0; 1.1)	0.05	0.8 (0.0; 1.5)	0.04

Notes: * Mean weight gain one year after smoking cessation for men from CAROSS study, aged 47 years (the cohort mean), smoking 10 cigarettes per day at baseline (the cohort minimum), having fewer than 20 weeks of smoking abstinence during the follow-up. **^†^** Weight gain increment with abstinence between 20 and 40 weeks with respect to the reference. **^‡^** Weight gain increment with more than 40 weeks abstinence with respect to the reference. **^§^** Difference in weight gain of women with respect to men, for 10 cigarettes smoked per day at baseline. **^||^** √NCB = squared root of the number of smoked cigarettes per day at baseline. **^#^** Effect of √NCB on weight gain for men. ** Additional effect of √NCB on weight gain for women (the effect of √NCB for women is 0.2 + 0.5 = 0.7, *p* = 0.002).

**Figure 3:**
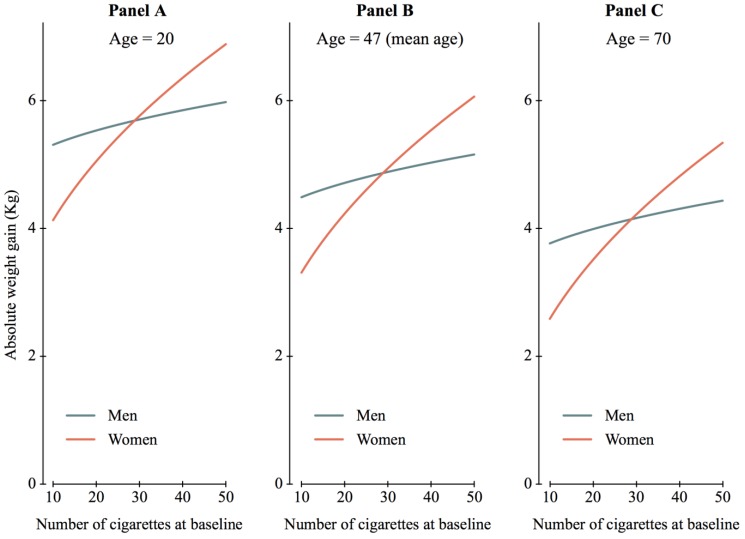
Mean absolute weight gain according to the multivariate model as a function of age, sex and number of cigarettes smoked at baseline. Overall duration of abstinence more than 40 weeks.

Age was also significantly associated with weight change, as weight gain was less with increasing age (*p* = 0.02): weight gain decreased by 0.3 kg (95% CI: 0.0 to 0.6) for each additional 10 years. We found a significant interaction between gender and the number of cigarettes smoked at baseline (Table 3 and Figure 3). When smoking 10 cigarettes at baseline, weight gain was less important for women than men (−1.2 kg, 95% CI: −2.2 to −0.2). However, the effect of the number of cigarettes on weight gain was more important for women (0.7 kg of additional gain per unit increment in the squared number of cigarettes; *p* = 0.002) than for men (only 0.2 kg of additional gain per unit increment in the squared number of cigarettes; *p* = 0.32), leading to a reversal of the gender effect at around 25 cigarettes smoked at baseline (Figure 3). The latter corresponds to the threshold sometimes adopted to define heavy smoking [28,29]. As a consequence, the subgroup at highest risk for weight gain was young women smoking heavily at baseline; they gained almost 7 kg on average 1 year after smoking cessation (Figure 3A).

In a similar model of weight gain relative to the initial weight, the results were comparable (Table 3) with a significant interaction between gender and the number of cigarettes. Among relatively light smokers, men gained more weight on average than women relative to their initial weight; the reverse was observed for heavy smokers.

## 4. Discussion

Using data from two RCTs assessing novel interventions aiming to help smokers quit, we found that weight gain was 2.6 kg (SD = 3.7) after 1 year of follow-up among smokers who attempted to quit. Weight gain increased according to duration of abstinence, reaching 4.6 kg (SD = 3.8) for abstinence of more than 40 weeks. Younger subjects gained significantly more weight than older subjects. We found an interaction between gender and amount of smoking at baseline; on average, women gained less weight than men when smoking fewer than 25 cigarettes per day at baseline, but the opposite was true for heavy female smokers. The amount of smoking at baseline was not significantly related to weight gain in men; they gained between 4.5 and 5.2 kg on average when smoking 10 to 50 cigarettes per day at baseline. In contrast, the amount of smoking was a strong predictor of weight gain for women, whose average weight gain ranged from 3.3 to 6.1 kg when smoking 10 to 50 cigarettes per day at baseline.

Our estimate of a mean weight gain of 4.6 kg (SD = 3.8) for subjects abstinent for more than 40 weeks (around 1 year) was consistent with the mean weight gain of 4.7 kg at 12 months (95% CI: 4.0 to 5.4) estimated by a recent meta-analysis of 62 studies [10]. Both estimates were much larger than earlier results of a mean weight gain between 1.8 and 3.6 kg at 1 year [8,9]. Earlier studies were based on the notion of point prevalence abstinence, which does not capture the individual abstinence-relapse trajectories and probably leads to an underestimation of weight gain [4,15]. The concept of overall duration of abstinence used here in order to estimate weight gain at 1 year is more consistent with the notion of continuous abstinence adopted in the more recent studies included in the meta-analysis, leading to comparable results. An advantage of using overall duration of abstinence is that we could identify a gradient in weight gain according to the overall time spent being abstinent instead of simply dichotomizing those who were abstinent for the entire period and those who were not (continuous abstinence). This result confirmed our hypothesis of a dose-response relationship between abstinence duration and weight gain. Adjusting for the cumulative duration of abstinence, we confirmed the negative effect of age on weight gain found by several authors [16,17,18] and found an explanation for the apparently contradictory results obtained in the literature regarding the effect of gender on weight gain [17,19,20,21,22]. We found that gender did not influence weight gain *per se*, but interacted with the amount of smoking before quitting; among subjects smoking relatively few cigarettes per day, men gained more weight on average than women, but among heavy smokers women gained more weight on average than men after quitting.

Our study was based on individual participant data from two RCTs. The combination was possible due to the multiple similarities between the two studies and because the interventions did not show any significant effect on smoking cessation rates, allowing us to merge individual data regardless of the RCT-specific randomization group. The joint use of two data sets allowed us to considerably improve the power of analyses, especially in the multivariable model; the same model estimated separately for each RCT gave similar results, but with less significance. However, merging the two RCTs required some assumptions. In CAROSS, almost all subjects were followed until the last 1-year visit, whereas the drop-out rate at the 1-year follow-up was approximately one-third in PHASMO. We assumed that subjects lost to follow-up had relapsed smoking [14], and that their weight stayed approximately stable once they left the study. Though empirically confirmed by a strong similarity between the weight gain distributions of the two RCTs, this assumption represents a limitation of the precision of our estimate of the mean weight gain at 1 year. A second limitation of our study is the small predictive power of the multivariate model (*R*^2^ = 0.21). According to the model, age, sex, amount of smoking before quitting, and duration of abstinence play major roles in weight gain after smoking cessation. However, if the aim is to predict weight gain after smoking cessation, other potentially confounding factors should be explored, such as nutrition, food intake, and physical activity. These data were not available for subjects in the RCTs.

## 5. Conclusions

In summary, we estimated that weight gain 1 year after smoking cessation was 4.6 kg (SD = 3.8 kg) among subjects who were abstinent for approximately a year. This result is consistent with estimates obtained by a recent comprehensive meta-analysis [10]. In addition, we found a strong and significant weight gain gradient according to the time of exposure to the abstinence state. We also found that younger ages are associated with more weight gain, and we highlighted a significant joint action of gender and the amount of smoking at baseline in explaining weight gain 1 year after cessation. Our results indicate a subgroup at increased risk of gaining weight: young women who smoked heavily before cessation. These patients should be followed up more closely by their physicians in regarding to their weight when they stop smoking.
